# PhlG mediates the conversion of DAPG to MAPG in *Pseudomonas fluorescens* 2P24

**DOI:** 10.1038/s41598-020-60555-9

**Published:** 2020-03-09

**Authors:** Ming-Min Zhao, Ning Lyu, Dong Wang, Xiao-Gang Wu, Yuan-Zheng Zhao, Li-Qun Zhang, Hong-You Zhou

**Affiliations:** 1 College of Horticulture and Plant Protection, Inner Mongolia Agricultural University, Hohhot, Inner Mongolia 010019 China; 2 College of Agriculture, Guangxi University, Nanning, 530004 China; 3 Key Laboratory of Plant Pathology, Ministry of Agriculture, Beijing, 100193 China; 4 Inner Mongolia Academy of Agricultural and Animal Husbandry Sciences, Hohhot, Inner Mongolia 010031 China

**Keywords:** Antimicrobials, Applied microbiology

## Abstract

The antibiotic 2,4-diacetylphoroglucinol (2,4-DAPG), produced by the Gram-negative rod-shaped bacterium *Pseudomonas fluorescens* 2P24, is active against various soil-borne bacterial and fungal pathogens that cause plant diseases. Biosynthesis of 2,4-DAPG is controlled by regulating expression of the *phlACBD* operon at the post-transcriptional level. The *phlG* gene is located between the *phlF* and *phlH* genes, upstream of the *phlACBD* biosynthetic operon. Herein, we cloned the *phlG* gene, generated a *phlG* deletion mutant, and investigated its regulatory role in 2,4-DAPG biosynthesis. The results showed that deletion of *phlG* had no effect on the biosynthesis of 2,4-DAPG, but it affected conversion of 2,4-DAPG to its precursor monoacetylphloroglucinol (MAPG). The global regulatory factor encoded by *gacS* positively regulated expression of *phlG*, while *rsmE* negatively regulated its expression. Deleting *phlG* did not alter the ability of the bacterium to colonise plants or promote plant growth. These results suggest that *phlG* collaborates with other factors to regulate production of the antibiotic 2,4-DAPG in *P. fluorescens* 2P24.

## Introduction

The antibiotic 2,4-diacetylphloroglucinol (DAPG) is produced by several *Pseudomonas* sp., including 2P24, CHA0, Pf-5, and YGJ3, and it plays a key role in inhibiting the growth of pathogenic microorganisms surrounding the plant rhizosphere^1–4^. As a phenolic secondary metabolite, 2,4-DAPG from some bacteria above has shown the capacity to control various plant pathogens. For example, *P. fluorescens* CHA0 protects plants against tobacco black root rot, *P. fluorescens* F113 protects sugar beet against *Pythium* damping-off, and *P. fluorescens* 2P24 protects against tomato bacterial wilt and wheat take-all diseases^5–11^. To further improve its potential applications, chemically synthesised 2,4-DAPG analogues have been developed and tested against plant diseases, and MP4, one of analogue of 2,4-DAPG, exhibited particularly potent antifungal activity, with inhibition rates of 84% and 63% against *Penicillium. digitatum* and *Penicillium. italicum*, respectively, and lower toxicity toward human cells compared with a fungicide widely used to treat harvested citrus fruit^12^. Such discoveries may assist the utilisation of DAPG analogues as novel biological fungicides for controlling plant diseases.

In *P. fluorescens*, the DAPG locus contains the four biosynthetic genes *phlACBD* that together produce 2,4-DAPG. PhlA, PhlC and PhlB are required for transacetylation of the monoacetylphloroglucinol (MAPG) precursor to generate DAPG^13,14^, and PhlD is critical for the biosynthesis of MAPG. In *P. fluorescens* 2P24, multiple factors in the GacS/GacA two-component system are involved in the biosynthesis of 2,4-DAPG during the late exponential and stationary phases^2,15,16^. The small RNA-binding proteins RsmA and RsmE, the resistance-nodulation-division efflux pump EmhABC, and the sigma factors RpoD, RpoN and RpoS are also associated with 2,4-DAPG biosynthesis^1,17–21^. In addition, PsrA is also a regulator of a sigma factor and involves in 2,4-DAPG biosynthesis PsrA negatively regulates *phlA* expression via either direct binding to an operator in the PhlA promoter region, or post-transcriptionally by affecting RpoS and RsmA expression. Inactivation of PsrA leads to a significant increase in 2,4-DAPG biosynthesis.

The *phlG* gene is also present in the DAPG biosynthetic locus of *P. fluorescens* 2P24, located between *phlF* and *phlH*. The PhlG protein is a DAPG hydrolase that may also modulate DAPG production^10^. The three-dimensional structure of PhlG revealed that the enzyme converts DAPG into MAPG by cleaving the carbon-carbon bond that attaches the acetyl group to the phenolic ring, hence PhlG differs functionally from classical α/β-hydrolases in terms of catalytic mechanism and substrate specificity^10^. Expression of PhlG is controlled by the pathway-specific regulators PhlF and PhlH, and *phlH* and *PhlG* impose negative feedback regulation on 2,4-DAPG biosynthesis^21^.

To further investigate the function of PhlG in the synthesis and metabolism of 2,4-DAPG in *P. fluorescens* 2P24, we performed thin-layer chromatography (TLC) and liquid chromatography (LC) assays to analyse 2,4-DAPG degradation, and generated and characterised PhlG mutants. The results confirm that PhlG converts DAPG to MAPG, and *rsmE* and *gacS* negatively or positively regulate *phlG* gene expression.

## Results

### Deletion of PhlG does not affect the growth of *P. fluorescens* 2P24

**Figure 1:**
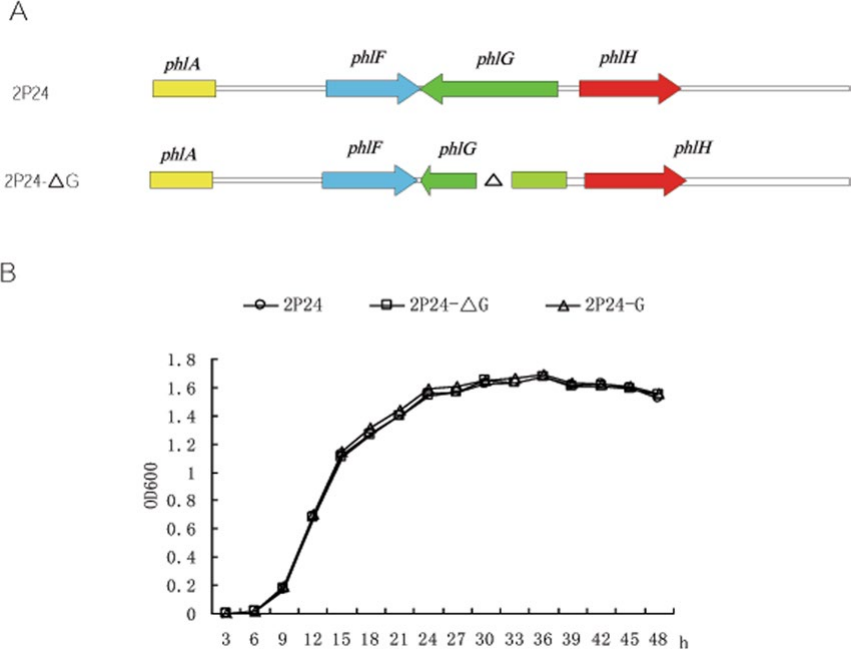
(**A**) Schematic diagram of the construction of the *phlG* deletion mutant in *Pseudomonas fluorescens* 2P24 and PM901. (**B**) Growth rate of wild-type (WT) *P. fluorescens* 2P24 and the *phlG* deletion mutant.

To investigate the influence of the *phlG* gene on the growth of *P. fluorescens* 2P24, the *PhlG* deletion (2P24-ΔG) and complementary line (2P24-G) were generated and transformed into 2P24 and PM901 as shown in Fig. 1A. The bacteria growth of 2P24, 2P24-ΔG and 2P24-G were evaluated at different time points (Fig. 1B). The results showed that the growth rate of the deletion mutant 2P24-ΔG was comparable with that of the WT and complementary strain 2P24-G, suggesting that deletion of *phlG* did not affect bacterial cell growth.

### PhlG mediates the conversion of DAPG to MAPG

**Figure 2:**
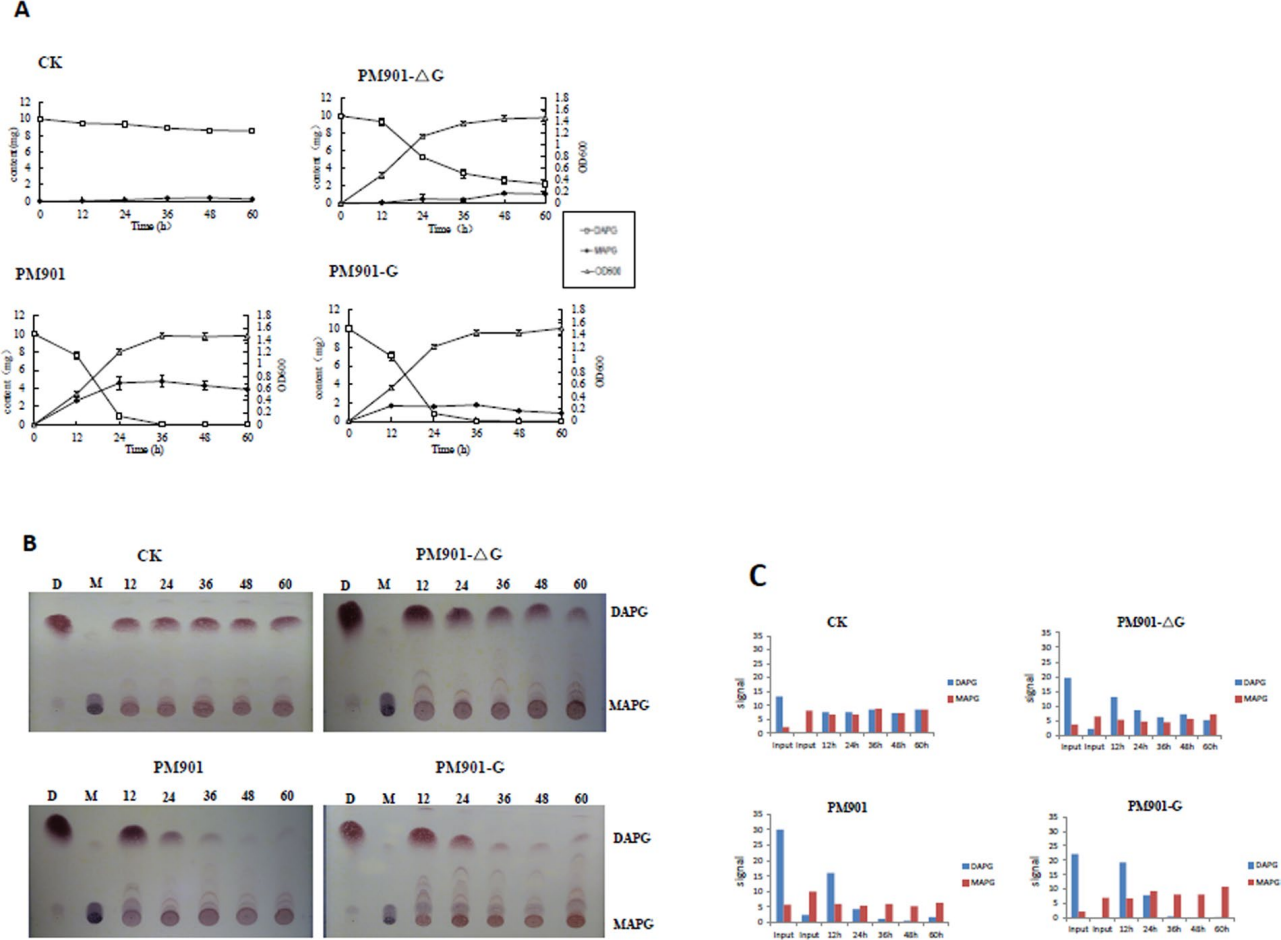
(**A**) Requirement of PhlG for the conversion of DAPG to MAPG. Strains PM901, PM901-ΔG (PhlG mutant), and PM901-G (PhlG complemented mutant) were grown at 27 °C in media supplemented with 100 μM DAPG, and bacterial cell growth and the concentrations of DAPG and MAPG were measured at the indicated time periods. Values are means and standard deviations from three independent cultures, and all experiments were performed in triplicate. **(B)** Degradation of 2,4-DAPG is regulated by PhlG. D, 2,4-DAPG at start of experiment; M, MAPG at start of experiment; measurements were made at 12, 24, 36, 48, and 60 h after inoculation. CK: Degradation profile of 2,4-DAPG without any treatment in 60 h. The original pictures of gels are shown in the supplementary information Fig. S3. (**C**) The signals of Fig. 3B from the original picture (Fig. S3) were quantified by Quantity ONE software.

A previous report showed that strain CHA0 degrades the potent antimicrobial agent DAPG to the much less toxic MAPG^14^. To investigate whether PhlG is also involved in this function in *P. fluorescens* 2P24, we deleted the *phlG* gene in strain PM901, a derivative of 2P24 in which *phlA* is deleted, rendering the strain unable to produce DAPG and MAPG. TLC and LC assays were performed to examine the potential regulatory role of PhlG in 2,4-DAPG biosynthesis. The culture of PM901(the *phlA* mutant, Δ*phlA*), PM901-ΔG (the *phlA* and *phlG* double mutant, Δ*phlA*Δ*phlG*) and PM901-G (the *phlG* complementary strain) were incubated in the presence (100 μM) and absence of DAPG. The results showed that the timing of DAPG reduction and MAPG increase is quite similar. With the PM901 strain, the DAPG concentration rapidly declined and the compound was undetectable after 48 h of incubation (Fig. 2A and Table S2). However, the DAPG concentration was decreased from 10 mg to 2 mg in the culture medium of PM901-ΔG strain lacking PhlG after 60 h incubation. When the PhlG is complemented in PM901-G strain, the accumulation of DAPG is similar to those of PM901, whereas the MAPG concentration increased rapidly from 12 h and was maintained at an elevated level at 60 h.

**Figure 3:**
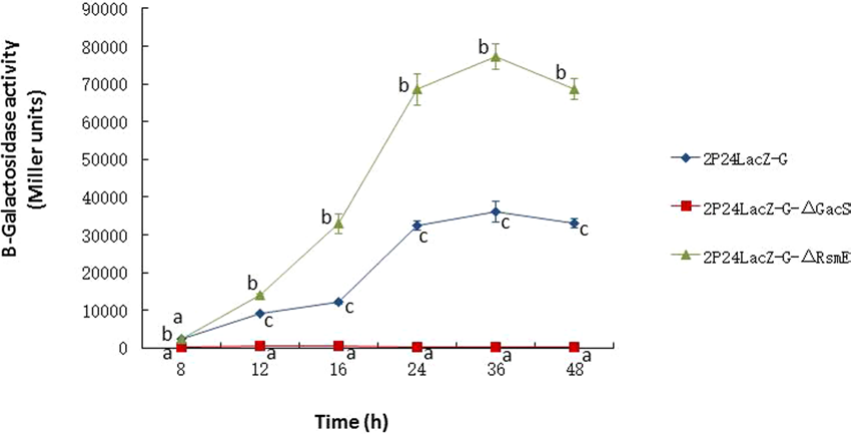
GacS and RsmZ regulate *phlG* expression in *P. fluorescens* 2P24.

Consistent with Fig. 3A, the results of TLC assays showed that DAPG degradation after 24 h was accompanied by the temporary accumulation of MAPG in the PM901 culture medium (Fig. 2B,C). In PM901-ΔG, the accumulation of DAPG is remained at higher level after 24 h and decreased at 60 h. By the contrast, the formation of MAPG increased from 24 h and showed high accumulation at 60 h. When PhlG was complemented in PM901-G, the patterns of DAPG and MAPG were similar to those of PM901. This suggested that PhlG is functional in conversion of DAPG to MAPG in *P. fluorescens* 2P24.

### *GacS* and *RsmZ* regulate *phlG* expression in *P. fluorescens* 2P24

In the biocontrol strain *P. fluorescens* CHA0, posttranscriptional repression of GacS/GacA-controlled genes relies highly on the RNA-binding protein RsmE and RsmA^21–23^. To explore whether DAPG biosynthesis is regulated by the *GacS/GacA* regulatory cascade through affecting the PhlG expression, *gacS* and *rsmE* genes in 2P24-LacZ-G were mutated and β-galactosidase activity was measured. We found that expression of *phlG* was completely inhibited when *gacS* gene was deleted, whereas *phlG* expression was markedly increased following deletion of *rsmE* (Fig. 3 and Table S3). Taken together, these results indicate that the global regulatory factor *gacS* positively regulates expression of *phlG*, while *rsmE* negatively regulates its expression.

### PhlG does not affect root colonization or plant growth in *P. fluorescens* 2P24

**Figure 4:**
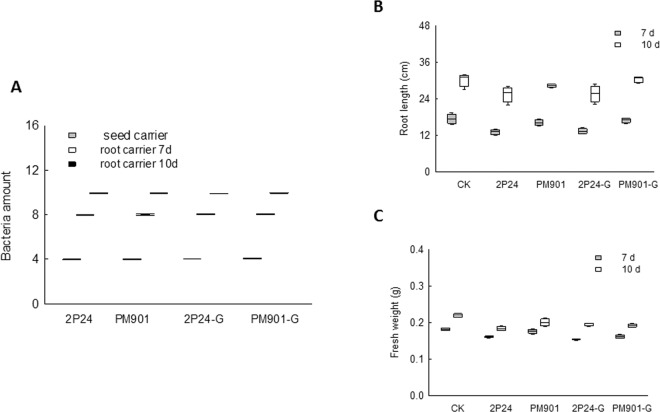
The effect of phlG mutation on the colonization capacity of 2P24 and the wheat growth. (**A)** *phlG* gene mutation does not change the colonization capacity of *P. fluorescens* 2P24. The values represent the number of bacteria carried on roots and seeds. The bacteria carrying on roots and seeds when treated with 2P24, PM901, 2P24-ΔG, PM9014-ΔG was represented by box plot. **(B)** *phlG* gene mutation does not promote wheat growth in length. The length of wheat plant when treated with 2P24, PM901, 2P24-ΔG, PM9014-ΔG was shown by box plot. **(C)** *phlG* gene mutation does not promote wheat growth in length. The weight of wheat plant when treated with 2P24, PM901, 2P24-ΔG, PM9014-ΔG was represented by box plot. CK: control plant, no treatment.

In order to investigate whether the colonisation ability of the 2P24 strain is influenced by *phlG*, the seed germination rate was determined after soaking seeds in cultures of strains 2P24, PM901, 2P24-ΔG and PM901-ΔG. The number of bacteria present on seeds and roots was measured at 7 d and 10 d post-sowing. As shown in Fig. 4A and Table S4, deletion of *phlG* did not alter the colonisation capacity. The effects of strains 2P24, PM901, 2P24-ΔG and PM901-ΔG on plant growth were also examined. There was no significant difference in plant height and root length when roots were colonised by 2P24-ΔG, PM901-ΔG and the respective WT strains (Fig. 4B,C and Table S5). This indicates that PhlG does not affect root colonization or plant growth in *P. fluorescens* 2P24.

## Discussion

2,4-DAPG is a key secondary metabolite in the biocontrol bacterium *Pseudomonas fluorescens* that inhibits the growth of pathogenic microorganisms in the plant rhizosphere. Our data showed that PhlG does not affect the antifungal activities of 2P24 against *Rhizoctonia solani* Kühn, which is different with the role of PhlG in CHA0^5^.

The protein encoded by the *phlG* gene catalyses the conversion of DAPG into the much less toxic MAPG by cleaving a carbon-carbon bond linking an acetyl group to the phenolic ring^24^. In this study, we confirmed the function of the product of the *phlG* gene that is located in the DAPG biosynthetic cluster in *P. fluorescens* 2P24, which is consistent with that phlG might also be associated with the DAPG biosynthetic locus in *P. fluorescens* strains Pf-5, Q2-87, F113 and CHA0^3,8,10,13,14^.

Together with a previous report in *P. fluorescens* CHA0^10^, our results of mutation of the *phlG* gene and DAPG degradation assays are underling that PhlG probably encodes a hydrolase that converts DAPG to MAPG. This suggests that PhlG-mediated degradation of DAPG is a conserved feature among DAPG-producing pseudomonads. In addition, we also found that deletion of *phlG* slowed down but did not eliminate degradation of 2,4-DAPG, suggesting other factors involoved in the DAPG degradation in this bacterium. MAPG is a direct precursor of DAPG biosynthesis^10,14,25^. We therefore propose that MAPG is also a degradation product of DAPG generated by PhlG, although the biological significance of this conversion remains controversial. PhlG appears to act on both the DAPG metabolite itself, and the DAPG biosynthetic operon. For example, accumulation of MAPG was increased in the *phlA* deletion mutant. By converting DAPG to the much less toxic MAPG, PhlG may help to prevent accumulation of the toxic metabolite, as demonstrated in strains CHA0 and F113^10^.

In *P. fluorescens*, the GacS/GacA system positively regulated the transcription of noncoding small RNAs, which further bind with repressor proteins RsmA and RsmE and release them from their target mRNAs^22,23^. The GacS mutant was used to investigate whether PhlG is regulated by the GacS/GacA system in *P. fluorescens*, and expression of *phlG* gene was markedly decreased when *gacS* was deleted, suggesting that this global regulatory factor positively regulates *phlG* expression. This further suggests that DAPG biosynthesis is highly dependent on the GacS/GacA regulatory cascade^14,16^. In *P. fluorescens* CHA0, the RNA-binding protein RsmA is a key regulatory element in the GacS/GacA signal transduction pathway that acts at the posttranscriptional level. The 64 amino acid polypeptide RsmE is a homolog of RsmA in strain CHA0, and RsmA and RsmE function together to cause maximal repression in the GacS/GacA cascade in this organism^21^. Deletion of the *rsmE* gene significantly up-regulated the expression of *phlG* in *P. fluorescens* 2P24, suggesting the negative regulation of RsmE on PhlG-mediated DAPG degradation. Additionally, PhlG expression is also negatively regulated by PhlF, a known pathway-specific transcriptional repressor of DAPG gene expression^21^.

Theoretically, mutant 2P24-∆G should be more inhibitory to the bacterial or fungal pathogens than 2P24, because it produces more DAPG. However, it appears that PhlG-mediated DAPG degradation does not affect either the root colonisation or plant growth-promoting activity of *P. fluorescens* 2P24. In *P. fluorescens* strains CHA0 and Pf-5, DAPG biosynthesis is negatively affected by pyoluteorin^14,26,27^, we thus speculate that other unknown factors probably mediate the antifungal activity of *P. fluorescens* 2P24 in the absence of PhlG. This means PhlG might have no direct effect on the synthesis of 2,4-DAPG; instead, phlG regulates the metabolism of 2,4-DAPG by assisting the conversion of DAPG to MAPG, which indicating that the deletion of phlG may have an indirect effect on the total amount of DAPG in the cell.

## Materials and Methods

### Bacterial strains, plasmids, and growth conditions

Bacterial strains and plasmids used in this study are listed in Table S1. *E. coli* and *P. fluorescens* were cultured as described previously in^28^.

### Plasmid construction and transformation

To generate the *phlG* deletion mutant p299ΔphlG, fragments flanking the *phlG* gene were amplified with four pair of primers using 2P24 genomic DNA as template. Forward primer Ga (5′-GGC*GGTACC*TACCGGTCAGCATGTG-3′) and reverse primer Gb (5′-CAG*GGATCC*CTGCGAGCTGGGC-3′) were used to amplify the left flanking sequence of the *phlG* gene, and the right flanking sequence was amplified with forward primer Gc (5′-GA*GGATCC*GGTTGAGGTCTTC-3′) and reverse primer Gd (5′-GTC*AAGCTT*CTGGAGAGACGATCGG-3′). After digestion with the relevant restriction enzymes, PCR fragments Gab and Gcd were cloned into pHSG299 and pHSG399 (TaKaRa), respectively. After digestion of p399ΔGcd with *Hind* III and *BamH* I, the excised fragment was ligated into the corresponding plasmid p299ΔphlG, and the two fragments were ligated at the *BamH* I site, resulting in the p299ΔphlG mutant, which resulted in the sequence of 317 bp (247–567) of PhlG gene (GenBank: DQ083928.1) was deleted (Fig. S1). The construct was verified by diagnostic PCR by perimer pair of G1/G2 and Ga/Gd using 2P24 genomic DNA and plasmid p299△G (Fig. S1A, B). We found that 300 bp fragment from both diagnosis PCR was lost in p299△G.

To generate the complementation plasmid, the coding region of the *phlG* gene was amplified by PCR with primer pair G1 (5′- AT*GAATTC*CCGTCATTGTCCCTTTAC -3′) and G2 (5′-AT*GTCGAC*ACTTCTGCTGAACGG-3′). The amplified fragment was digested and ligated with plasmid pRK415 to generate recombinant plasmid p415-phlG referenced to Wei *et al*., 2005 and Wu *et al*., 2012, which was transformed into 2P24 and PM901, resulting in strains 2P24-G and PM901-G.

Plasmid p299-phlG and 3 kb *LacZ* gene fragments were digested with *BamH* I, and ligated to create p299-LacZ-G. The correct plasmid was transferred to the strain 2P24. The homologous recombination mutant 2P24-LacZ-G was screened X-gal-containing LB plates. Using strains 2P24-LacZ-G and 2P24-ΔG as templates, using primer G1: 5′-ATGTCGACACTTCTGCTGAACGG-3′ and G2: 5′-ATGTCGACACTTCTGCTGAACGG-3′ were subjected to PCR examination (Fig. S2C).

To obtain *gacS* gene from *P. fluorescens* 22P4, the chromosomal DNA of *P. fluorescens* 22P4 was digested with *Mob* l and ligated to pLAFRS with *BamH* l and *Sac* I. Then, it was transfected into *E. coli* DH5a after *in vitro* packaging to construct a genomic library. According to the published sequence of *gacS* gene, primers S-H: 5′-TCGGCATCAACCGCATGGC-3′ and S-G: 5′-GTGCCTTCGCGGGTGAACTT-3′ in the conserved region were designed. PCR amplification was performed using the strain 2P24 genome DNA as a template. The resulting fragment (0.54 kb) was verified by sequencing, and the 2P24 genomic library was screened by PCR using this as a marker. The verified PCR containing 3.2 kb fragment and 2.5 kb was shown in Fig. S2D. The resulting positive fragment was sub-cloned and ligated into plasmid pBluescript and verified by sequencing.

The construction of 2P24ΔGacS is referenced to Hailei Wei and Liqun Zhang^29^. Based on the obtained *gacS* gene sequence from 2P24 strain, primer S1: 5′-GATAAGCTTGGCAGCACTC-3′ and S3186: 5′- ATGGATCCAGCTTAACCGC-3′ were designed. PCR amplification was carried out using the strain 2P24 genome as a template. The PCR product was digested with the corresponding enzyme and ligated with pBluescript to construct the recombinant plasmid pS-BH. pS-BH was digested with *EcoR* I and self-ligated to obtain the recombinant vector pS-BEH. The The pS-BEH was digested with *Sal* l and *BamH* I, and ligated with the corresponding digested pSR47s to construct a suicide vector (pSR47s△S). The pSR47s△S was transformed into 2P24 by the method of parental hybridization, and the recombinant bacteria were screened by using kanamycin as a marker. The selected recombinant bacteria were cultured at 28°C for 36 h without antibiotic pressure, and continuously transferred to culture for 4 times. The strains which lost the kanamycin resistance were screened, and the second recombinant strain was produced, thereby obtaining the deletion mutant (2P24ΔGacS).

The *rsmE* gene of *P. fluorescens* 2P24 was found in the genome sketch of *P. fluorescens* 2P24 by homology alignment according to the sequence of other *rsmE* genes of *P. fluorescens* that have been reported. The left flanking fragment (1.015 kb) was amplified by primers RsmE-2285 (5′-ATC TGCAGAAGGGCCAGTACGGCTC-3′) and RsmE-3330 (5′-ATGGATCC TATGAGTGGGCGTTTCAGCC- 3′) using 2P24 genome DNA as template. After digestion with *Pst* I and *BamH* I, the fragment was ligated into the corresponding digested pHSG399 to obtain p399RsmE (L) and confirmed by sequencing. The primers RsmE-3498 and RsmE-4685 were designed for PCR amplification using the 2P24 strain genome as a template. The right flanking fragment (1.108 kb) was amplified with primers RsmE-3498 (5′- ATGGATCC TCACCGCCCCGACAAGCCGC - 3′) and RsmE-4658 (5′- CGGTGCTGTTCGAAATGGTGCG - 3′) using 2P24 genome DNA as template. The same digestion strategy was followed as used in p399RsmE (L) construction. Then, the plasmid p399RsmE (L) was cultured and digested with *Pst* I. The digested fragment was recovered and then ligated into the corresponding digested pHSG399RsmE(R), and the resulting vector was named pHSG399ΔRsmE.

The pHSG399ΔRsmE was digested with *EcoR* I-*Sac* I. The fragment was recovered and then ligated into the corresponding digested pBSKm. The resulting vector was named pBSKmΔRsmE. The vectors containing *GacS* and *RsmE* mutant were introduced into the mutant 2P24-LacZ-G by electroporation, and the two-step homologous recombination mutants lacking the *GacS* and *RsmE* genes were screened by PCR (Fig. S2E). The β-galactosidase enzyme activity of the mutant strain was simultaneously determined.

### Measurement of β-galactosidase activity

The procedure for the construction of reporter fusions and measurement of β-galactosidase activity is described in Wu *et al*. (2012).

### Determination of DAPG and MAPG production by TLC and LC assays

Strains were cultured overnight and adjusted to an absorbance at 600 nm (OD_600)_ of 0.8 with LB. Control (CK) cultures were not mock inoculated. Next, 10 mg of 2,4-DAPG dissolved in methanol was added after 10 h and cultures were incubated at 28 °C with shaking at 150 rpm for 12, 24, 36, 48, or 60 h. Cells were centrifuged at 7000 *g* for 10 min and the supernatant was acidified to pH 2.0 with 1 M HCl. The organic phase was extracted by rotary evaporation with an equal volume of ethyl acetate, and the dry solid was dissolved in 0.15 mL of methanol. Samples were spotted on the bottom of a silicone plate, dried with a sterilised airflow, and separated by TLC using a solvent system comprised of chloroform:acetone (19:1 v:v). A 1–5% ferric chloride ethanol solution spray was applied for visualised of the developed plate. LC was used to assess 2,4-DAPG and MAPG standards.

### Root colonisation test and wheat growth assay

Wheat seeds of uniform size without visible wounds were selected and used to investigate the *in vivo* biological activities of 2P24-ΔG and PM901-ΔG. Wild-type (WT) 2P24 and PM901 strains were included as controls. Strains 2P24, PM901, 2P24-ΔG and PM901-ΔG were separately inoculated into 5 mL of LB medium and incubated at 28 °C with shaking at 150 rpm for 24 h. When the culture concentration reached an OD_600_ value of 0.056 or 5×10^7^ colony forming units (cfu)/mL, wheat seeds were soaked inside the culture for overnight. The wheat seeds were planted into a pot containing 30 g mixture of meteorite and soil. Three seeds per pot and 3 pots per treatment were prepared. At 7 days and 10 days, soil was gently removed from roots by shaking, and roots were placed in test tubes containing 10 mL of sterile water and agitated at 150 rpm for 10 min. The number of bacteria in the test tube was calculated, and the root length and plant fresh weight were determined. All tests were repeated for three times.

### Statistical analysis

GraphPad Prism software version 5.01 (Graphpad Software, Inc.) was used for analysis of variance, followed by multiple comparisons using one way ANOVA, and *p *< 0.05 was considered statistically significant.

## Supplementary information

Supplementary Figures

Legends of Supplementary Figures

Supplementary Table S1

Supplementary Table S2

Supplementary Table S3

Supplementary Table S4

Supplementary Table S5
