# Supplementary material for: PhlG mediates the conversion of DAPG to MAPG in Pseudomonas fluorescens 2P24
Source: Sci Rep. 2020 Mar 9;10:4296. doi: 10.1038/s41598-020-60555-9 (PMC7062750; doi:10.1038/s41598-020-60555-9)
Supplement: Supplementary file 1 — Supplementary Figures [file 41598_2020_60555_MOESM1_ESM.pdf]

Title： PhlG mediates the conversion of DAPG to MAPG in *Pseudomonas fluorescens* 2P24

Author list： Zhao Ming-min[1†], Lyu Ning[1†], Wang Dong[1], Wu Xiao-gang[2], Zhao Yuan-zheng [4], Zhang Li-qun[2,3] and Zhou Hong-you [1*]

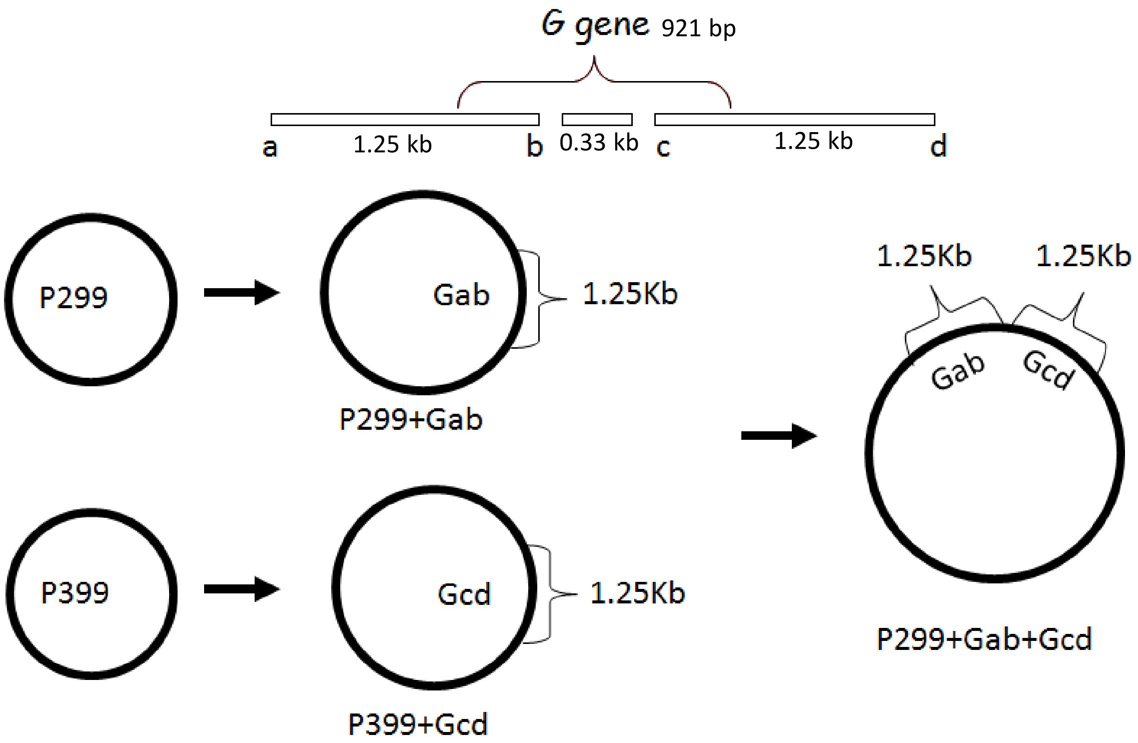

Figure S1 Schematic diagram of plasmid construction

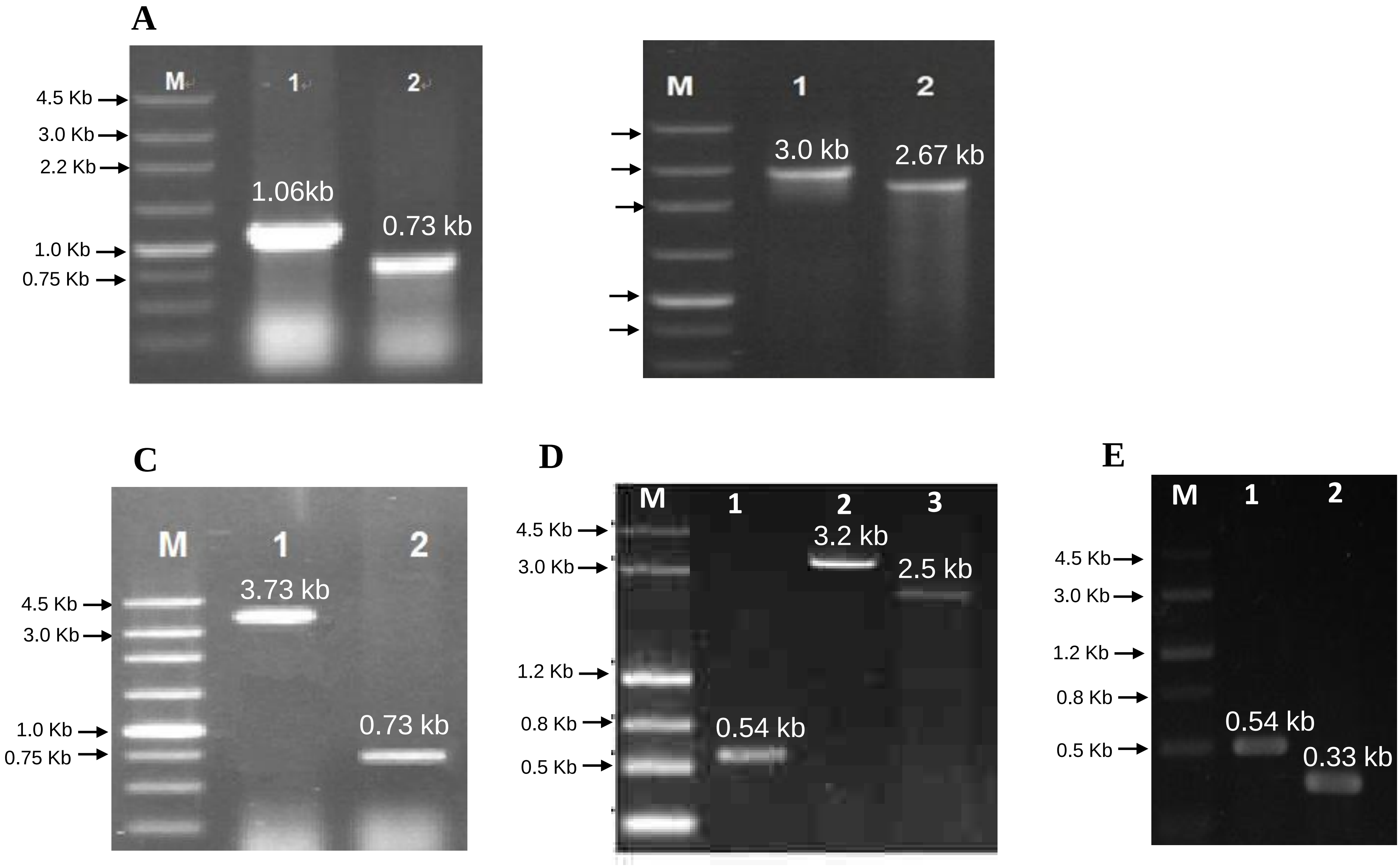

Figure S2 The diagnostic PCR to confirm mutants

CK

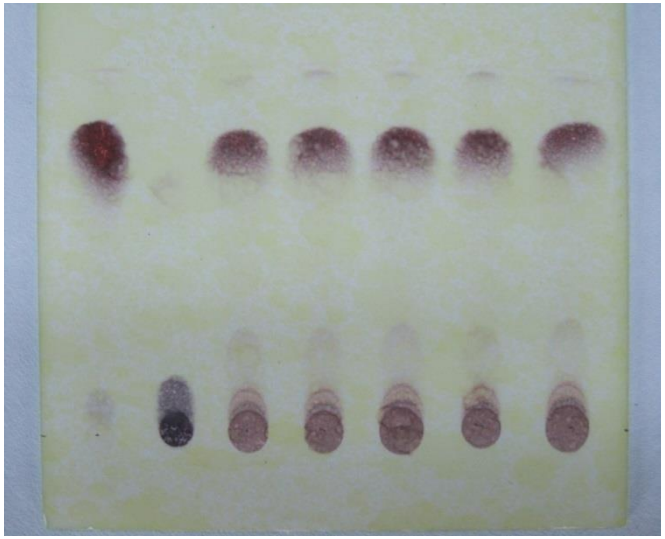

PM901

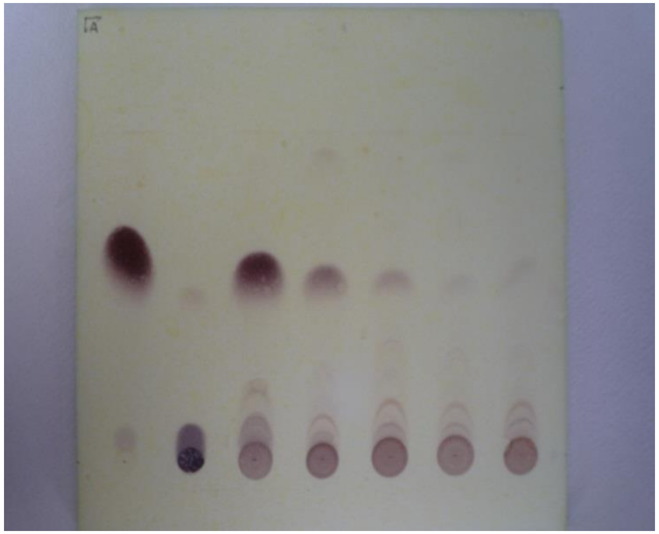

PM901-G

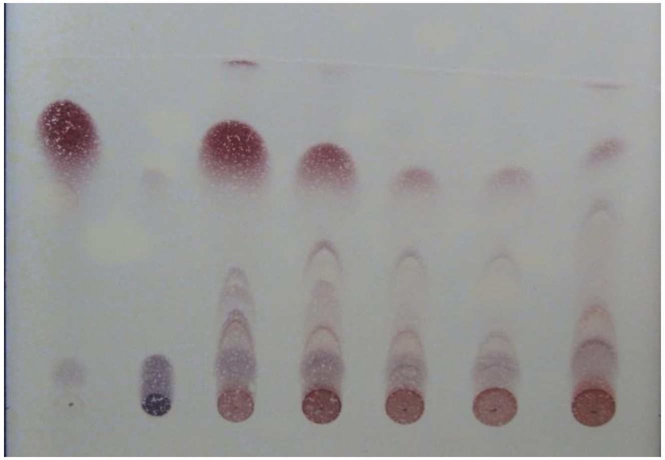

PM901-△G

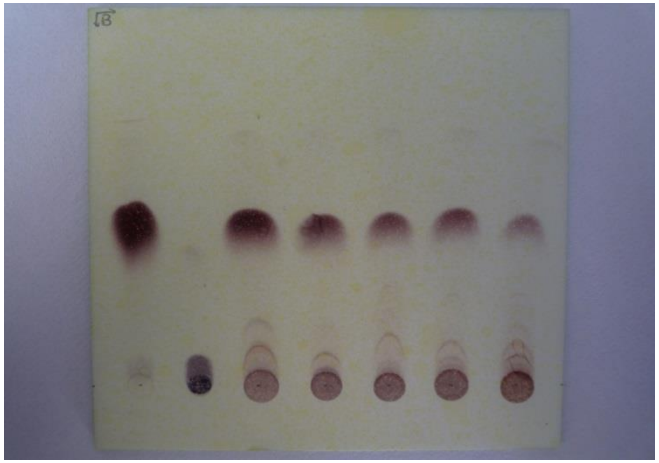

Figure S3 Original pictures of Figure 3B
