# Supplementary material for: PhlG mediates the conversion of DAPG to MAPG in Pseudomonas fluorescens 2P24
Source: Sci Rep. 2020 Mar 9;10:4296. doi: 10.1038/s41598-020-60555-9 (PMC7062750; doi:10.1038/s41598-020-60555-9)
Supplement: Supplementary file 2 — Legends of Supplementary Figures [file 41598_2020_60555_MOESM2_ESM.pdf]

Title: PhlG mediates the conversion of DAPG to MAPG in *Pseudomonas fluorescens* 2P24

Author list: Zhao Ming-min[1 †], Lyu Ning[1 †], Wang Dong[1], Wu Xiao-gang[2], Zhao Yuan-zheng [4], Zhang Li-qun[2,3] and Zhou Hong-you [1*]

Figure S1 Schematic diagram of plasmid construction

To generate the *phlG* deletion mutant p299ΔphlG, fragments flanking the *phlG* gene were amplified with four pair of primers using 2P24 genomic DNA as template. The left flanking sequence and the right flanking sequence of the *phlG* gene were amplified. After digestion with the relevant restriction enzymes, PCR fragments Gab and Gcd were cloned into pHSG299 and pHSG399 (TaKaRa), respectively. The excised fragment of p399ΔGcd digested by *Hind*III and *BamH*I was ligated into the corresponding plasmid p299ΔphlG. The two fragments were ligated at the *BamH*I site to create p299ΔphlG mutant.

Figure S2 The diagnostic PCR to confirm mutant genotypes

(A) PCR detection of recombinant plasmid p299△G using primer G1/G2.

1: the fragment (1.06 kb) amplified from 2P24 genomic DNA by PCR; 2: the fragment (0.75 kb) amplified from p299△G. **(B)**PCR detection of recombinant plasmid p299△G using primer Ga/Gd. M: marker(1kb DNA Ladder marker,TaKaRa）; 1: the fragment (3.0 kb) amplified from 2P24 genomic DNA by PCR; 2: the fragment (2.67 kb) amplified from p299△G. **(C)** PCR detection of recombinant plasmid 2P24-LaZ-G. 1: the fragment (3.73 kb) amplified from 2P24-LacZ-G by PCR; 2: the fragment (0.73 kb) amplified from 2P24- △G. **(D)** PCR detection of the 2P24ΔGacS.1: the Control 0.54 kb fragment; 2: the fragment (3.2 kb) amplified from 2P24- △GacS; 3: the fragment (2.5 kb) amplified from 2P24- △GacS. **(E)** PCR detection of the 2P24-ΔRsmE. 1: the fragment (0.54 bp) amplified from 2P24; 2: the fragment (0.33 bp) amplified from 2P24-ΔRsmE.

Figure S3 Original pictures of Figure 3B

The original pictures of figure 3B are shown and used for making the quantification by Quantity ONE software for figure 3C.
