# Supplementary material for: PhlG mediates the conversion of DAPG to MAPG in Pseudomonas fluorescens 2P24
Source: Sci Rep. 2020 Mar 9;10:4296. doi: 10.1038/s41598-020-60555-9 (PMC7062750; doi:10.1038/s41598-020-60555-9)
Supplement: Supplementary file 3 — Supplementary Table S1 [file 41598_2020_60555_MOESM3_ESM.pdf]

Title: PhlG mediates the conversion of DAPG to MAPG in *Pseudomonas fluorescens* 2P24

Author list: Zhao Ming-min[1 †], Lyu Ning[1 †], Wang Dong[1], Wu Xiao-gang[2], Zhao Yuan-zheng[4], Zhang Li-qun[2,3] and Zhou Hong-you [1*]

Table S1 The bacterial strains and plasmids used in this study

Strains or plasmids	Characteristics	Source
**Strains**		
P. fluorescens 2P24	Wild-type, Ap^r	Wei et al., 2005
E. Coli DH5α	F- recA1 endA1 hsdR17 deoR thi-1 supE44 gyrA96 relA1△ (lacZYA-argF)U169λ-(Φ80dlacz △ M15)	Stratagene, La Jolla, CA
PM901	Derivative of *P. fluorescens* 2P24, *phlA-lacZ* reporter fusion, Ap^r	Zhou et al., 2010
2P24-△G	*phlG* deletion mutant, Ap^r	This study
2P24-G	2P24-△G with plasmid p415-phlG, Ap^r, Tc^r	This study
2P24-LacZ-G	Derivative of *P. fluorescens* 2P24, *phlG-lacZ* reporter fusion, Ap^r	This study
2P24-LacZ-G-△GacS	*GacS* deletion mutant, Ap^r	This study
2P24-LacZ-G-△RsmE	*RsmE* deletion mutant, Ap^r	This study
PM901-G	PM901-△G with plasmid p415-phlG, Ap^r, Tc^r	This study
CPF-10	Wild-type, Sm^r, Ap^r	Wu et al., 2012
S-16	Wild-type, Sm^r	Wu et al., 2012
Rhizoctonia solani	*Causing cotton damping-off*	This study
**Plasmids**		
pHSG299	Suicide plasmid for *Pseudomonas* spp., used for homologous recombination, Km^r	TaKaRa
pHSG399	Suicide plasmid for *Pseudomonas* spp., used for homologous recombination, Cm^r	TaKaRa
pRK415	Broad-host-rang cloning vector; IncP1 replicon; polylinker of pUC19; Mob+ ; Tet^r	Keen, *et al.*
P415-phlG	pRK415 containing intact *phlG* gene, Tc^r	This study
p299-△phlG	pHSG299 containing *phlG* deletion structure, Km^r	This study
pSR47s△GacS	pSR47s containing *GacS* deletion structure, Km^r	Wei et al., 2004
pBSKm△RsmE	pBSKm containing RsmE deletion structure, Kmr	Liu et al., 2011

Note: Km^r ,Cm^r , Tc^r, Sm^r and Ap^r indicate resistance to ampicillin, kanamycin, chloramphenicol and tetracycline
