# Supplementary material for: PhlG mediates the conversion of DAPG to MAPG in Pseudomonas fluorescens 2P24
Source: Sci Rep. 2020 Mar 9;10:4296. doi: 10.1038/s41598-020-60555-9 (PMC7062750; doi:10.1038/s41598-020-60555-9)
Supplement: Supplementary file 6 — Supplementary Table S4 [file 41598_2020_60555_MOESM6_ESM.pdf]

Title： PhlG mediates the conversion of DAPG to MAPG in *Pseudomonas fluorescens* 2P24

Author list： Zhao Ming-min[1 †], Lyu Ning[1 †], Wang Dong[1], Wu Xiao-gang[2], Zhao Yuan-zheng[4], Zhang Li-qun[2,3] and Zhou Hong-you [1*]

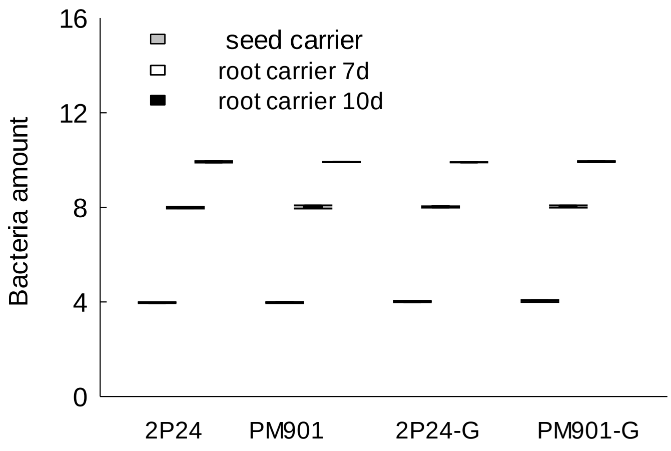

Seed carrier

LSD

Treatment(I)	treatment(J)	Mean Difference (I-J)	Std. Error	Sig.	95% Confidence Interval	
					Lower Bound	Upper Bound
2P24	PM901	-0.00874	0.017835	0.633086	-0.04759	0.030122
	2P24-△G	-0.03997	0.017835	0.044716	-0.07883	-0.00111
	PM901-△G	-0.06862	0.017835	0.002319	-0.10748	-0.02977
PM901	2P24	0.008736	0.017835	0.633086	-0.03012	0.047594
	2P24-△G	-0.03123	0.017835	0.10541	-0.07009	0.007627
	PM901-△G	-0.05989	0.017835	0.005695	-0.09875	-0.02103
2P24-△G	2P24	0.039968	0.017835	0.044716	0.001109	0.078826
	PM901	0.031232	0.017835	0.10541	-0.00763	0.07009
	PM901-△G	-0.02866	0.017835	0.134072	-0.06752	0.010201
PM901-△G	2P24	0.068625	0.017835	0.002319	0.029766	0.107483
	PM901	0.059889	0.017835	0.005695	0.02103	0.098747
	2P24-△G	0.028657	0.017835	0.134072	-0.0102	0.067516

* The mean difference is significant at the 0.05 level.

2P24 c

PM901 bc

2P24-△G ab

PM901-△G a

Root carrier 7d

LSD

treatment(I)	treatment(J)	Mean Difference (I-J)	Std. Error	Sig.	95% Confidence Interval	
					Lower Bound	Upper Bound
2P24	PM901	-0.04011	0.0261	0.150265	-0.09698	0.016755
	2P24-△G	-0.03823	0.0261	0.168691	-0.0951	0.018637
	PM901-△G	-0.05263	0.0261	0.066697	-0.1095	0.004237
PM901	2P24	0.040111	0.0261	0.150265	-0.01675	0.096977
	2P24-△G	0.001882	0.0261	0.943702	-0.05498	0.058748
	PM901-△G	-0.01252	0.0261	0.640126	-0.06938	0.044348
2P24-△G	2P24	0.038229	0.0261	0.168691	-0.01864	0.095095
	PM901	-0.00188	0.0261	0.943702	-0.05875	0.054984
	PM901-△G	-0.0144	0.0261	0.591265	-0.07127	0.042466
PM901-△G	2P24	0.052629	0.0261	0.066697	-0.00424	0.109495
	PM901	0.012518	0.0261	0.640126	-0.04435	0.069384
	2P24-△G	0.0144	0.0261	0.591265	-0.04247	0.071266

* The mean difference is significant at the 0.05 level.

2P24 a

PM901 a

2P24-△G a

PM901-△G a

Root carrier 10d

LSD

Treatment(I)	treatment(J)	Mean Difference (I-J)	Std. Error	Sig.	95% Confidence Interval	
					Lower Bound	Upper Bound
2P24	PM901	0.012854	0.011261	0.275933	-0.01168	0.037389
	2P24-△G	0.019299	0.011261	0.112247	-0.00524	0.043833
	PM901-△G	0.002292	0.011261	0.842099	-0.02224	0.026827
PM901	2P24	-0.01285	0.011261	0.275933	-0.03739	0.011681
	2P24-△G	0.006445	0.011261	0.577653	-0.01809	0.03098
	PM901-△G	-0.01056	0.011261	0.366781	-0.0351	0.013973
2P24-△G	2P24	-0.0193	0.011261	0.112247	-0.04383	0.005236
	PM901	-0.00644	0.011261	0.577653	-0.03098	0.01809
	PM901-△G	-0.01701	0.011261	0.156851	-0.04154	0.007528
PM901-△G	2P24	-0.00229	0.011261	0.842099	-0.02683	0.022242
	PM901	0.010562	0.011261	0.366781	-0.01397	0.035096
	2P24-△G	0.017006	0.011261	0.156851	-0.00753	0.041541

* The mean difference is significant at the 0.05 level.

2P24 a

PM901 a

2P24-△G a

PM901-△G a
