# Supplementary material for: PhlG mediates the conversion of DAPG to MAPG in Pseudomonas fluorescens 2P24
Source: Sci Rep. 2020 Mar 9;10:4296. doi: 10.1038/s41598-020-60555-9 (PMC7062750; doi:10.1038/s41598-020-60555-9)
Supplement: Supplementary file 7 — Supplementary Table S5 [file 41598_2020_60555_MOESM7_ESM.pdf]

Title: PhlG mediates the conversion of DAPG to MAPG in *Pseudomonas fluorescens* 2P24

Author list: Zhao Ming-min[1 †], Lyu Ning[1 †], Wang Dong[1], Wu Xiao-gang[2], Zhao Yuan-zheng[4], Zhang Li-qun[2,3] and Zhou Hong-you [1*]

We performed multiple comparison by the one-way Anova analysis and LSD to test the differences.

Length 7d

LSD

treatment(I)		Mean Difference (I-J)	Std. Error	Sig.	95% Confidence Interval Lower Bound	95% Confidence Interval Upper Bound
CK	2P24	4.32500*	0.74956	0.000	2.7274	5.9226
	PM109	1.22500	0.74956	0.123	-0.3726	2.8226
	2P24-ΔG	4.10000*	0.74956	0.000	2.5024	5.6976
	PM109-ΔG	0.62500	0.74956	0.417	-0.9726	2.2226
2P24	CK	-4.32500*	0.74956	0.000	-5.9226	-2.7274
	PM109	-3.10000*	0.74956	0.001	-4.6976	-1.5024
	2P24-ΔG	-0.22500	0.74956	0.768	-1.8226	1.3726
	PM109-ΔG	-3.70000*	0.74956	0.000	-5.2976	-2.1024
PM901	CK	-1.22500	0.74956	0.123	-2.8226	.3726
	2P24	3.10000*	0.74956	0.001	1.5024	4.6976
	2P24-ΔG	2.87500*	0.74956	0.002	1.2774	4.4726
	PM109-ΔG	-0.60000	0.74956	0.436	-2.1976	0.9976
2P24-ΔG	CK	-4.10000*	0.74956	0.000	-5.6976	-2.5024
	2P24	0.22500	0.74956	0.768	-1.3726	1.8226
	PM109	-2.87500*	0.74956	0.002	-4.4726	-1.2774
	PM109-ΔG	-3.47500*	0.74956	0.000	-5.0726	-1.8774
PM109-ΔG	CK	-0.62500	0.74956	0.417	-2.2226	0.9726
	2P24	3.70000*	0.74956	0.000	2.1024	5.2976
	PM109	0.60000	0.74956	0.436	-0.9976	2.1976
	2P24-ΔG	3.47500*	0.74956	0.000	1.8774	5.0726

* The mean difference is significant at the 0.05 level.

CK a

2P24 b

PM901 a

2P24-ΔG b

PM109-ΔG a

Length 10d

LSD

treatmen(I)	treatment(J)	Mean Difference (I-J)	Std. Error	Sig.	95% Confidence Interval	
					Lower Bound	Upper Bound
CK	2P24	4.50000*	1.40101	0.006	1.5138	7.4862
	PM901	1.52500	1.40101	0.294	-1.4612	4.5112
	2P24-ΔG	4.35000*	1.40101	0.007	1.3638	7.3362
	PM109-ΔG	-0.37500	1.40101	0.793	-3.3612	2.6112
2P24	CK	-4.50000*	1.40101	0.006	-7.4862	-1.5138
	PM901	-2.97500	1.40101	0.051	-5.9612	0.0112
	2P24-ΔG	-0.15000	1.40101	0.916	-3.1362	2.8362
	PM109-ΔG	-4.87500*	1.40101	0.003	-7.8612	-1.8888
PM901	CK	-1.52500	1.40101	0.294	-4.5112	1.4612
	2P24	2.97500	1.40101	0.051	-0.0112	5.9612
	2P24-ΔG	2.82500	1.40101	0.062	-0.1612	5.8112
	PM109-ΔG	-1.90000	1.40101	0.195	-4.8862	1.0862
2P24-ΔG	CK	-4.35000*	1.40101	0.007	-7.3362	-1.3638
	2P24	0.15000	1.40101	0.916	-2.8362	3.1362
	PM901	-2.82500	1.40101	0.062	-5.8112	0.1612
	PM109-ΔG	-4.72500*	1.40101	0.004	-7.7112	-1.7388
PM109-ΔG	CK	0.37500	1.40101	0.793	-2.6112	3.3612
	2P24	4.87500*	1.40101	0.003	1.8888	7.8612
	PM901	1.90000	1.40101	0.195	-1.0862	4.8862
	2P24-ΔG	4.72500*	1.40101	0.004	1.7388	7.7112

* The mean difference is significant at the 0.05 level.

CK a

2P24 b

PM901 ab

2P24-ΔG b

PM109-ΔG a

Weight 7d

LSD

treatmen(I)	treatment(J)	Mean Difference (I-J)	Std. Error	Sig.	95% Confidence Interval	
					Lower Bound	Upper Bound
CK	2P24	0.06250*	0.00907	0.000	0.0432	0.0818
	PM901	0.01700	0.00907	0.080	-0.0023	0.0363
	2P24-ΔG	0.03275*	0.00907	0.003	0.0134	0.0521
	PM109-ΔG	0.04700*	0.00907	0.000	0.0277	0.0663
2P24	CK	-0.06250*	0.00907	0.000	-0.0818	-0.0432
	PM901	-0.04550*	0.00907	0.000	-0.0648	-0.0262
	2P24-ΔG	-0.02975*	0.00907	0.005	-0.0491	-0.0104
	PM109-ΔG	-0.01550	0.00907	0.108	-0.0348	0.0038
PM901	CK	-0.01700	0.00907	0.080	-0.0363	0.0023
	2P24	0.04550*	0.00907	0.000	0.0262	0.0648
	2P24-ΔG	0.01575	0.00907	0.103	-0.0036	0.0351
	PM109-ΔG	0.03000*	0.00907	0.005	0.0107	0.0493
2P24-ΔG	CK	-0.03275*	0.00907	0.003	-0.0521	-0.0134
	2P24	0.02975*	0.00907	0.005	0.0104	0.0491
	PM901	-0.01575	0.00907	0.103	-0.0351	0.0036
	PM109-ΔG	0.01425	0.00907	0.137	-0.0051	0.0336
PM109-ΔG	CK	-0.04700*	0.00907	0.000	-0.0663	-0.0277
	2P24	0.01550	0.00907	0.108	-0.0038	0.0348
	PM901	-0.03000*	0.00907	0.005	-0.0493	-0.0107
	2P24-ΔG	-0.01425	0.00907	0.137	-0.0336	0.0051

* The mean difference is significant at the 0.05 level.

CK a

2P24 c

PM901 ab

2P24-ΔG b

PM109-ΔG bc

Weight 10d

LSD

treatmen(I)	treatment(J)	Mean Difference (I-J)	Std. Error	Sig.	95% Confidence Interval	
					Lower Bound	Upper Bound
CK	2P24	0.03350*	0.00688	0.000	0.0188	0.0482
	PM901	0-.00250	0.00688	0.721	-0.0172	0.0122
	2P24-ΔG	0.02675*	0.00688	0.001	0.0121	0.0414
	PM109-ΔG	0.03575*	0.00688	0.000	0.0211	0.0504
2P24	CK	-0.03350*	0.00688	0.000	-0.0482	-0.0188
	PM901	-0.03600*	0.00688	0.000	-0.0507	-0.0213
	2P24-ΔG	-0.00675	0.00688	0.342	-0.0214	0.0079
	PM109-ΔG	0.00225	0.00688	0.748	-0.0124	0.0169
PM901	CK	0.00250	0.00688	0.721	-0.0122	0.0172
	2P24	0.03600*	0.00688	0.000	0.0213	0.0507
	2P24-ΔG	0.02925*	0.00688	0.001	0.0146	0.0439
	PM109-ΔG	0.03825*	0.00688	0.000	0.0236	0.0529
2P24-ΔG	CK	-0.02675*	0.00688	0.001	-0.0414	-0.0121
	2P24	0.00675	0.00688	0.342	-0.0079	0.0214
	PM901	-0.02925*	0.00688	0.001	-0.0439	-0.0146
	PM109-ΔG	0.00900	0.00688	0.210	-0.0057	0.0237
PM109-ΔG	CK	-0.03575*	0.00688	0.000	-0.0504	-0.0211
	2P24	-0.00225	0.00688	0.748	-0.0169	0.0124
	PM901	-0.03825*	0.00688	0.000	-0.0529	-0.0236
	2P24-ΔG	-0.00900	0.00688	0.210	-0.0237	0.0057

* The mean difference is significant at the 0.05 level.

CK a

2P24 b

PM901 a

2P24-ΔG b

PM109-ΔG b
